# A biological phenotype of suicide attempt in adolescents with nonsuicidal self-injury: a machine-based learning approach

**DOI:** 10.1038/s41386-025-02176-2

**Published:** 2025-07-29

**Authors:** Erik Fink, Corinna Reichl, Stefan Lerch, Julian Koenig, Michael Kaess

**Affiliations:** 1 University Hospital of Child and Adolescent Psychiatry and Psychotherapy, University of Bern, Bern, Switzerland; 2 Graduate School for Health Sciences, University of Bern, Bern, Switzerland; 3 University of Cologne, Faculty of Medicine and University Hospital Cologne, Department of Child and Adolescent Psychiatry, Psychosomatics and Psychotherapy, Cologne, Germany; 4 Department of Child and Adolescent Psychiatry, University Hospital Heidelberg, Heidelberg, Germany

**Keywords:** Predictive markers, Risk factors

## Abstract

Suicide attempts (SA) are a common risk in adolescents with non-suicidal self-injury (NSSI). In the present study, we investigated whether a set of biological markers contributed (above clinical features) to the distinction of adolescents with NSSI and SA from those with NSSI alone using machine-based learning approaches. Female adolescents engaging in NSSI (*n* = 161) were recruited from our outpatient clinic for risk-taking and self-harming behavior (AtR!Sk). Different machine-based learning models (logistic regression, elastic net regression, random forests, gradient boosted trees) with repeated cross-validation were applied. We tested whether a) the full set of neurobiological markers, b) a reduced set including preselected markers based on existing evidence (CRP, interleukin-6, salivary cortisol, DHEA-S, TSH, dopamine, norepinephrine, ACTH), and c) a model with only depressive symptoms and age could distinguish between the two groups (NSSI + SA vs. NSSI alone). Depressive symptoms and age were included as covariates in the reduced set to account for their potential predictive effects. The reduced set of neurobiological markers showed poor to fair predictive performance (AUC between 0.62 and 0.72) for SA depending on the model. Predictors with the highest predictive value were high DHEA-S (OR = 1.47, 95% CI = 1.04–2.09) and low TSH (OR = 0.68, 95% CI = 0.48–0.97). Complex models slightly outperformed simpler ones and feature selection modestly increased predictive performance. The study may suggest a future potential of biomarkers for the assessment of suicide risk among adolescents with NSSI. Further research is needed to replicate these findings longitudinally.

## Introduction

Self-harm, encompassing both suicide attempts (SA) and non-suicidal self-injury (NSSI), is a serious health issue among adolescents. Among 15–19-year-olds, suicide was the fourth most common cause of death between 2000 and 2019 worldwide [1]. Approximately 5–16% of adolescents in the general population report a lifetime SA [2], while 17–18% in the general population report NSSI [3]. NSSI typically begins around ages 12–13 years, peaking at 15–16 years [4] and decreases during late adolescence and young adulthood. In clinical populations, NSSI has been found to be associated with SA in ~30–40% of cases [5]. While NSSI can provide momentary relief from suicidal ideation due to its tension and stress-relieving properties [6], it is also one of the strongest predictors of SA, typically preceding SA by an average of 1.5 years [7–9]. Given that SA represents a serious adverse event in the treatment of mental health problems, it is crucial to identify adolescents at particularly high risk for SA within the risk group of adolescents engaging in NSSI.

However, identifying risk for SA within generally high risk adolescents is a particularly challenging task. Franklin et al. summarized five decades of suicide research in a meta-analysis, revealing that the prediction of SA by common risk factors is only slightly better than chance [10]. Furthermore, these challenges are even more evident within clinical samples [11]. Nevertheless, recent advances in machine learning [12, 13] may enhance prediction accuracy by incorporating diverse data types, including biomarkers. Machine learning could potentially overcome previous challenges in risk-stratification of self-harm, particularly by integrating biomarkers [14–16]. As part of these advancements, growing evidence has identified potential biomarkers of NSSI [17] as well as SA [18, 19], with notable overlaps given the close association of NSSI with SA.

Among the key biological systems studied in both types of self-harm, the endocrine stress response system—primarily involving the hypothalamic-pituitary-adrenal (HPA) axis—has gained significant attention. Changes in cortisol levels, a crucial HPA axis neurosteroid, in blood and saliva are associated with NSSI and SA [20–22]. Another important biomarker of the HPA axis is the adrenocorticotropic hormone (ACTH), which has been studied in stress-related disorders [23, 24].

Beyond the HPA axis, biomarkers of other biological systems show promise for predicting SA in NSSI. For instance, neurobiological markers of the hypothalamus-pituitary-gonadal (HPG) and hypothalamus-pituitary-thyroid (HPT) axes, such as the sex hormone dehydroepiandrosterone sulfate (DHEA-S) and thyroid-stimulating hormone (TSH), have been shown to be correlated with SA [25, 26] and NSSI [27]. Similarly, immunological markers, including interleukin-6 (Il-6) [28] and C-reactive protein (CRP) [29] have been linked to both NSSI [30] and suicide risk [31]. In addition, biomarkers of the autonomic nervous system (ANS), such as heart rate variability (HRV) [32], as well as dopamine and noradrenaline [33] may also aid in predicting SA in adolescents with NSSI. Evidence indicates altered pain processing in the context of self-harm [32, 34, 35], but research on the potential role of pain sensitivity in SA is still lacking. Furthermore, emerging research suggests that biological markers derived from neuroimaging [36–38] might contribute to identify adolescents with NSSI at risk for SA. In addition, decreased oxytocin levels in blood have been found in survivors of SA and might therefore predict suicide risk [39]. While the research findings summarized above mainly focused on associations between single biomarkers and self-harm, a previous study from our group has shown that a combined set of markers, namely oxytocin, DHEA-S, beta-endorphin, free triiodothyronine (fT3), leukocytes, HRV and pain sensitivity, was able to discriminate adolescents with NSSI from healthy controls (HC) using machine learning approaches [16].

Given the established links between these markers and NSSI, a similar or potentially different set of biomarkers might also be able to discriminate between adolescents who engage in NSSI and attempted suicide from those who only exhibit NSSI. Testing the full set of biomarkers may provide a comprehensive understanding, while focusing on a reduced set of markers may improve accuracy and practicality in clinical settings. In the reduced set, we focused on blood and saliva biomarkers selected for their practicality in clinical settings and their potential to identify adolescents with NSSI at high risk for SA. These biomarkers were chosen based on empirical support for associations with suicidal behavior, as highlighted in recent meta-analyses and systematic reviews [19, 20, 22, 25, 26].

The present study aims to investigate whether a) the full set of biomarkers versus b) the reduced set (CRP, interleukin-6, salivary cortisol, DHEA-S, TSH, dopamine, noradrenaline, and ACTH), which is known to be associated with suicidal behavior, and c) a model including only depressive symptoms and age, could distinguish between adolescents with NSSI and a history of SA (NSSI + SA) and those with NSSI without a history of SA using machine learning. Depressive symptoms were considered as a covariate in the reduced set to determine if there is a direct link between the biomarkers and SA, over and above depressive symptoms.

## Materials and methods

### Participants and procedure

All participants took part in a cohort study from the outpatient clinic for adolescent risk-taking and self-harming behavior (AtR!Sk; Ambulanz für Risikoverhalten und Selbstschädigung) at the Clinic for Child and Adolescent Psychiatry, Heidelberg University Hospital, Germany (ethical approval: S-449/2013). After the clinical diagnostic assessment performed by trained clinicians, patients were invited to a second appointment at which a neurobiological assessment took place (AtR!Sk-Bio study; ethical approval: S-514/2015). Only baseline cross-sectional data from patients who took part in the neurobiological assessment were eligible for inclusion in the present study. Inclusion criteria were written informed consent from patients and their caregivers, an age between 12 and 17 years, and proficiency in the German language. Exclusion criteria were pregnancy, acute psychosis, and underlying neurological, endocrinological, or cardiovascular disease. All participants received a reimbursement of €40 for participating in the study. Based on known sex-differences regarding the prevalence of NSSI and underlying biological systems, only female participants were included in our study.

### Biological assessment

Peripheral blood samples were collected from adolescents after an overnight fast. All samples were drawn between 8:30 and 9:00 a.m. to account for diurnal variations in hormone levels. After the blood draw, Salivettes (Sarstedt) collecting devices were used to measure salivary cortisol and amylase. Saliva sampling was followed by the assessment of resting HRV (rHRV) and resting functional near-infrared spectroscopy (rfNIRS). During these assessments, participants were engaged in a simple color detection task [40].

### Clinical assessment

During the clinical assessment, information about sex, medication, smoking, drug use and date of birth was obtained. NSSI and SA was assessed via the German version of the Self-Injurious Thoughts and Behaviors Interview (SITBI-G) [41, 42]. All participants met Criterion A of the DSM-5 research diagnosis for NSSI disorder. SA was assessed as a dichotomous variable distinguishing between adolescents who reported at least one lifetime suicide attempt and those who have not reported any past SA. Depressive symptoms were assessed via the Depression Inventory for Children and Adolescents Questionnaire (DIKJ) [43]. Additionally, psychiatric diagnoses were assessed using the Mini International Neuropsychiatric Interview for Children and Adolescents (Mini-Kid) [44].

### Biological measures

Biological markers (Table 1) were derived from blood samples, saliva samples, ECG recordings and rfNIRS.

**Table 1:** Biological markers.

Biological system	Biological marker	Measurement
ANS	Adrenaline	Blood sample
	**Dopamine**	Blood sample
	rHRV	ECG
	**Noradrenaline**	Blood sample
Emotion processing	Deoxyhemoglobin PFC	rfNIRS
	Oxyhemoglobin PFC	rfNIRS
	Oxytocin	Blood sample
	Total hemoglobin PFC	rfNIRS
HPA axis	**ACTH**	Blood sample
	Amylase	Saliva sample
	Blood cortisol	Blood sample
	**Salivary Cortisol**	Saliva sample
HPG axis	**DHEA-S**	Blood sample
	Estradiol	Blood sample
	Testosterone	Blood sample
HPT axis	fT3	Blood sample
	fT4	Blood sample
	**TSH**	Blood sample
Immune system	**CRP**	Blood sample
	Leukocytes	Blood sample
	**Il-6**	Blood sample
Pain processing	Beta-Endorphin	Blood sample

Biomarkers in bold were part of the reduced set.

*fT3* free triiodothyronine, *fT4* free thyroxine, *ACTH* adrenocorticotropic hormone, *CRP* C-reactive protein, *IL-6* Interleukin-6, *DHEA-S* dehydroepiandrosterone-sulfate, *rHRV* resting heart rate variability, *PFC* prefrontal cortex, *rfNIRS* resting functional near-infrared spectroscopy.

#### Blood samples

Blood sample analyses were conducted at the central laboratories of Heidelberg University Hospital in adherence to their accredited procedures. ACTH was defined in EDTA-plasma using a chemiluminescence immunoassay (CLIA). Thyroid function was assessed based on levels of TSH, fT3, and fT4 by an ADVIDA Centaur immunoassay. Blood Cortisol was assessed in serum through a CLIA process via the CENTAUR XPT® (Siemens Healthineers). For leukocytes, flow cytometry with the ADVIA2120® (Siemens Healthineers) on EDTA blood samples was used. CRP and estradiol were defined in Li-heparin-plasma through CLIA using the ADVIA XPT® chemistry device from Siemens Healthineers. IL-6 serum levels were measured through CLIA, employing the IMMULITE® 2000 XPI analyzer from Siemens. Beta endorphin and oxytocin levels were assessed using ELISA by Cloud Clone (Houston, TX, US) and DHEA-S using an in-house radioimmunoassay. Adrenaline, noradrenaline, and dopamine were assessed in EGTA plasma using a High-Performance Liquid Chromatography (HPLC) process with a HPLC system from VWR international. Testosterone levels were defined in Li-heparin plasma using an Electrochemiluminescence Immunoassay (ECLIA) using a Cobas e601, e411 device from Roche. Reference ranges can be obtained from the Supplementary.

#### Saliva samples

Until the assay, saliva samples were frozen and kept at −20 °C. Salivettes were centrifuged at 3000 rpm for 5 min after thawing, producing a clear supernatant with low viscosity. Using a highly sensitive chemiluminescence immunoassay that is commercially accessible, salivary concentrations of cortisol and amylase were assessed (IBL International, Hamburg, Germany). A liquid handling robot (Genesis, Tecan, Switzerland) was used for semi-automated sample and reagent handling, and quality control samples with low, medium, and high cortisol concentrations were run on each microtiter plate being tested.

#### NIRS

An 8-channel continuous-wave NIRS system (OctaMon, Artinis, The Netherlands) assessed PFC oxygenation and deoxygenation. This technique uses light sources and receivers affixed to participants’ foreheads, emitting light at 760 nm and 850 nm to detect oxygenated (O2Hb) and deoxygenated (HbR) hemoglobin, based on the modified Beer-Lambert law. For a detailed description of the preprocessing pipeline applied to this sample, refer to [37, 38, 45–47]. Final hemoglobin concentration values for O2Hb, HbR, and HbT were exported for analysis.

#### ECG

Resting HRV was assessed using an ECG Move III device (movisens GmbH, Karlsruhe, Germany), reflecting autonomic nervous system influence. For a detailed description of the preprocessing pipeline applied to this sample, refer to ref. [32, 46, 47]. The primary HRV metric was the root mean square of successive differences (rMSSD), indicating vagally-mediated HRV.

### Statistical analyses

This study aimed to apply machine learning models to distinguish adolescents with NSSI and SA from those with NSSI alone based on neurobiological markers. Data preparation and preprocessing were conducted in Stata (Version 18; StataCorp LP, College Station, TX, USA). To address missing data, multiple imputation by chained equations (MICE) was performed, resulting in 500 imputed datasets [48]. Predictive mean matching was used for imputation, and our model included the full set of biomarkers (Table 1), depressive symptoms, and age. This high number of imputations was selected to ensure robust and stable estimates. Rubin’s rules [49] were used to combine results across imputations, producing pooled estimates and standard errors.

Five predictor sets were tested: a) the full set of biomarkers, b) the reduced set, c) the reduced set with depressive symptoms included as a covariate, d) the reduced set with depressive symptoms and age included as covariates, and e) a model including only depressive symptoms and age as predictors. SA served as the outcome variable in all models. Four model types were fitted for each predictor set: logistic regression, elastic net regression, random forests, and gradient-boosted trees. These models span a range of analytical approaches; elastic net includes regularization for variable selection [50], while random forests [51] and gradient boosting [52] can capture complex interactions without assuming linearity.

To prevent overestimation in model performance, we used repeated five-fold cross-validation for internal validation, in which the data were repeatedly split into training and test sets (five folds), and this entire process was repeated twenty times to ensure stable estimates [53]. The primary performance metric was the area under the ROC curve (AUC). All model fitting and evaluation were conducted in R (Version 4.3.0 [54]), with cross-validation supported by the caret package (Version 6.0.94 [55]). The glmnet package (Version 4.1.7 [56]) was used for elastic net regression, ranger (Version 0.15.1 [57]) for random forests, and xgboost (Version 1.7.5.1 [52]) for gradient boosted trees.

Feature importance was assessed across the 500 imputed datasets. For elastic net and logistic regression models, importance was determined by the absolute values of coefficients, and we recorded the frequency with which each feature had the highest coefficient across imputations. For random forests and gradient boosted trees, approximate SHAP values (SHapley Additive exPlanations [58]) were calculated, identifying the most important predictor by highest SHAP value in each dataset. Stability of feature importance was evaluated by counting how often each predictor ranked as the top feature across imputations. Additionally, pooled odds ratios and confidence intervals were estimated in Stata to provide summary effect sizes across imputations. These estimates were obtained using a separate logistic regression model fitted to the reduced set, independent of the machine learning models used for classification.

## Results

Of the *n* = 242 patients enrolled in the AtR!Sk-Bio study, data from *n* = 61 patients who did not meet DSM-5 criteria for NSSI were excluded. In addition, *n* = 19 males and *n* = 2 patients outside the age range were excluded. Therefore, the final sample (*n* = 161) included *n* = 77 female adolescent patients with NSSI + SA and *n* = 84 female adolescent patients with NSSI only (Table 2).

**Table 2:** Sample characteristics.

	NSSI alone	NSSI + SA	Statistics
	(*n* = 84)	(*n* = 77)	
Age			
Mean (SD)	14.6 (1.38)	15.2 (1.48)	*t*_(159)_ = −2.99, *p* = 0.003, *d* = −0.47
Median [Min, Max]	14.0 [12.0, 17.0]	15.0 [12.0, 17.0]	
Adverse childhood experience			
Yes	48 (57.1%)	54 (70.1%)	*χ²*_*(*1,144)_ = 2.88, *p* = 0.09. Cramér’s *V* = 1
Missing	9 (10.7%)	8 (10.4%)	
Psychoactive medication			
Yes	4 (4.8%)	13 (16.9%)	*χ²*_(1,161)_ = 5.03, *p* = 0.025, Cramér’s *V* = 0.20
Drug consumption			
Yes	23 (27.4%)	29 (37.7%)	*χ²*_(1,161)_ = 1.5, *p* = 0.221, Cramér’s *V* = 0.11
Smoking			
Yes	40 (47.6%)	47 (61.0%)	*χ²*_*(*1,161)_ = 2.4, *p* = 0.122, Cramér’s *V* = 0.13
Depressive symptoms			
Mean (SD)	29.9 (9.49)	30.1 (8.51)	
Missing	10 (11.9%)	9 (11.7%)	
CRP [mg/l]			
Mean (SD)	2.42 (1.74)	3.90 (6.84)	
Missing	5 (6%)	5 (6.5%)	
Leukocytes [/nl]			
Mean (SD)	6.50 (1.59)	7.00 (2.46)	
Missing	8 (9.5%)	6 (7.8%)	
TSH [mU/l]			
Mean (SD)	2.39 (1.22)	1.89 (0.943)	
Missing	6 (7.1%)	4 (5.2%)	
fT3 [ng/l]			
Mean (SD)	3.42 (0.379)	3.43 (0.387)	
Missing	6 (7.1%)	4 (5.2%)	
fT4 [ng/l]			
Mean (SD)	11.7 (1.96)	11.7 (1.49)	
Missing	6 (7.1%)	4 (5.2%)	
Estradiol [pg/ml]			
Mean (SD)	88.5 (96.8)	79.7 (55.9)	
Missing	7 (8.3%)	5 (6.5%)	
Testosterone [ng/ml]			
Mean (SD)	0.27 (0.15)	0.36 (0.18)	
Missing	6 (7.1%)	5 (6.5%)	
ACTH [pg/ml]			
Mean (SD)	18.7 (9.46)	15.8 (9.79)	
Missing	8 (9.5%)	6 (7.8%)	
Il-6 [pg/ml]			
Mean (SD)	2.63 (3.74)	2.89 (2.55)	
Missing	10 (11.9%)	12 (15.6%)	
Blood Cortisol [ng/ml]			
Mean (SD)	174 (75.6)	157 (56.2)	
Missing	6 (7.1%)	8 (10.4%)	
DHEA-S [µg/ml]			
Mean (SD)	1.74 (0.931)	2.18 (1.18)	
Missing	7 (8.3%)	8 (10.4%)	
Noradrenaline [pmol/l]			
Mean (SD)	1890 (916)	1950 (3120)	
Missing	16 (19%)	14 (18.2%)	
Adrenaline [pmol/l]			
Mean (SD)	1030 (3860)	389 (409)	
Missing	16 (19%)	14 (18.2%)	
Dopamine [pmol/l]			
Mean (SD)	616 (499)	781 (829)	
Missing	16 (19.2%)	15 (19.5%)	
Oxytocin [µg/ml]			
Mean (SD)	125 (99.2)	135 (141)	
Missing	9 (10.7%)	11 (14.3%)	
Beta-Endorphin [pg/ml]			
Mean (SD)	33.8 (32.0)	35.6 (28.4)	
Missing	9 (10.7%)	11 (14.3%)	
Salivary Cortisol [nmol/l]			
Mean (SD)	3.20 (2.24)	3.58 (2.90)	
Missing	0 (0%)	1 (1.3%)	
Amylase [U/ml]			
Mean (SD)	190 (186)	158 (135)	
Missing	1 (1.2%)	2 (2.6%)	
rHRV (rMSSD)			
Mean (SD)	56.2 (30.3)	54.3 (28.4)	
Missing	4 (4.8%)	5 (6.5%)	
Oxyhaemoglobin PFC			
Mean (SD)	−0.303 (0.965)	−0.241 (0.807)	
Missing	1 (1.2%)	7 (9.1%)	
Desoxyhaemoglobin PFC			
Mean (SD)	−0.295 (0.670)	−0.267 (0.721)	
Missing	1 (1.2%)	7 (9.1%)	
Total-Haemoglobin PFC			
Mean (SD)	−0.598 (1.37)	−0.507 (1.16)	
Missing	1 (1.2%)	7 (9.1%)	
Diagnoses^a^			
F10–F19, *n* (%)	13 (15.4)	22 (28.6)	*χ²*_(1, 161)_ = 3.32, *p* = 0.069, Cramér’s *V* = 0.16
F30–F39, *n* (%)	56 (66.7)	46 (59.7)	*χ²*_(1, 161)_ = 0.56, *p* = 0.455, Cramér’s *V* = 0.07
F40–F48, *n* (%)	34 (40.5)	34 (44.2)	*χ²*_(1, 161)_ = 0.1, *p* = 0.755, Cramér’s *V* = 0.04
F50–F59, *n* (%)	10 (11.9)	10 (13)	*χ²*_(1, 161)_ = 0, *p* = 1, Cramér’s *V* = 0.02
F60–F69, *n* (%)	22 (26.2)	42 (54.6)	χ²_(1, 161)_ = 12.33, *p* = 0.001, Cramér’s *V* = 0.29
F80–F89, *n* (%)	0 (0)	1 (1.3)	*χ²*_(1, 161)_ = 0, *p* = 0.965, Cramér’s *V* = 0.08
F90–F98, *n* (%)	17 (20.2)	25 (32.5)	*χ²*_(1, 161)_ = 2.51, *p* = 0.113, Cramér’s *V* = 0.14

*SD* standard deviation, Smoking: on more than one day in the last year. Drug consumption: at least one day of drug consumption during the last year. *fT3* free triiodothyronine, *fT4* free thyroxine, *ACTH* adrenocorticotropic hormone, *CRP* C-reactive protein, Il-6 Interleukin-6, *DHEA-S* dehydroepiandrosterone-sulfate, *rHRV* resting heart rate variability, *PFC* prefrontal cortex, *NSSI* non-suicidal self-injury.

^a^No F0, F2, or F7 disorders were diagnosed.

Patients with NSSI + SA were slightly older than patients with NSSI alone (Table 2). Also, a larger proportion of patients with NSSI + SA reported taking psychoactive medications. When examining psychiatric diagnoses, significant group difference emerged for F60–F69 diagnoses (personality disorders), indicating that a larger proportion of patients with both NSSI + SA were diagnosed with personality disorders compared to those with NSSI alone.

### Association between biomarkers and clinical outcome

#### The full set of biomarkers

**Fig. 1:**
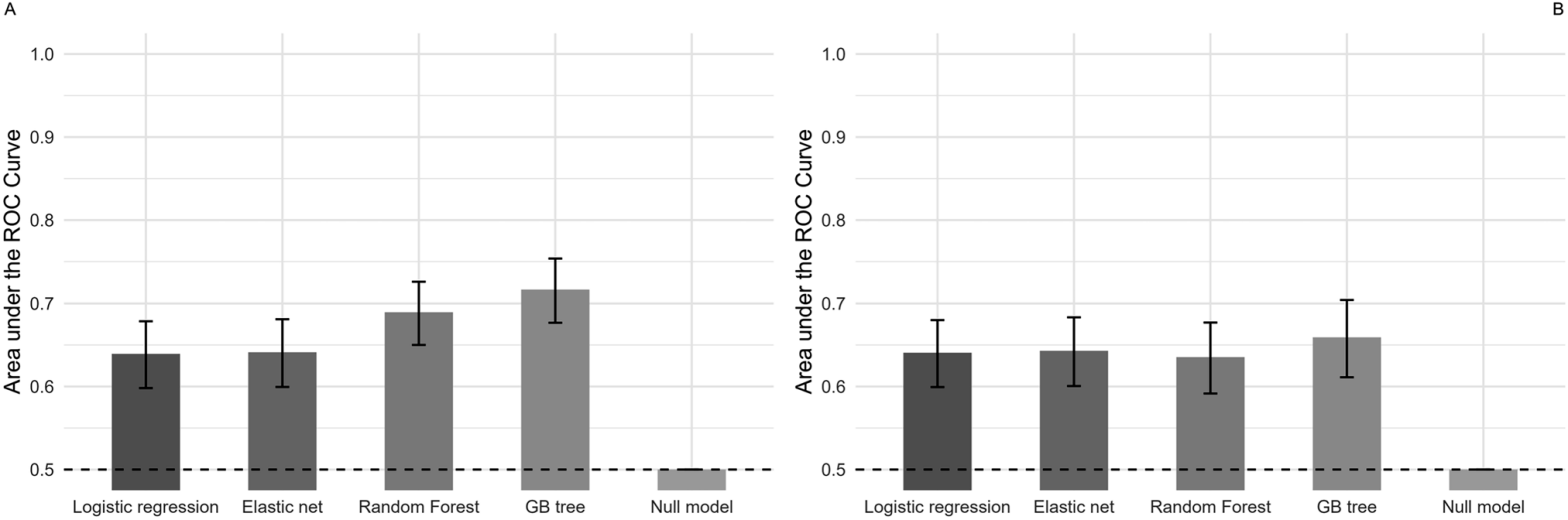
Area under the curve (AUC) for machine learning models discriminating between female patients with NSSI + SA and those with NSSI alone. Panel (**A**) includes the reduced set and panel (**B**) includes the full  set. The bars represent the performance of machine learning models: A model with AUC = 1 would be considered as perfect, 0.9 < AUC < 1 as excellent, 0.8 < AUC < 0.9 as good, 0.7 < AUC < 0.8 as fair, 0.6 < AUC < 0.7 as poor and 0.5 < AUC < 0.6 as being slightly better than chance. The error bars represent the 95% confidence intervals (CIs) of AUC values.

The machine learning models including the full set of biomarkers showed poor performance in discriminating between patients with NSSI + SA and those with NSSI alone (Fig. 1A and Table 3). Performance indices (AUC and the standard deviation of the AUC (AUCSD)) did not differ between the models (logistic regression, elastic net, random forest, gradient boosted tree). During the repeated cross-validation process, which consisted of five folds performed 20 times, the logistic regression model predicted that, on average, 55.0% of the observations were associated with the NSSI + SA group. In comparison, the proportions for the Elastic Net Regression, Random Forest, and Gradient-Boosted Tree models were 55.3%, 57.7%, and 54.13%, respectively.

**Table 3:** Area under the receiver operating characteristics curve, standard deviations and confidence intervals for models discriminating between female patients with NSSI + SA and those with NSSI alone using the full set, and the reduced set respectively.

	Models including the full set				Models including the reduced set			
Model	AUC	AUCSD	LB	UB	AUC	AUCSD	LB	UB
Logistic regression	0.641	0.089	0.599	0.860	0.639	0.089	0.598	0.679
Elastic net	0.643	0.092	0.600	0.863	0.641	0.090	0.599	0.681
Random forest	0.635	0.094	0.592	0.677	0.690	0.096	0.650	0.726
Gradient-boosted tree	0.659	0.110	0.611	0.704	0.717	0.096	0.677	0.754

A model with AUC = 1 would be considered as perfect, 0.9 < AUC < 1 as excellent, 0.8 < AUC < 0.9 as good, 0.7 < AUC < 0.8 as fair, 0.6 < AUC < 0.7 as poor and 0.5 < AUC < 0.6 as being slightly better than chance. Lower (LB) and upper bounds (UB) of the 95% confidence intervals (CIs) of AUC values are reported.

#### The reduced set of biomarkers

Machine learning models including the reduced set of biomarkers showed poor to fair performance in discriminating between adolescents with NSSI + SA and those with NSSI alone (Fig. 1B and Table 3). More complex models (gradient-boosted trees and random forests) performed slightly better than linear models (logistic regression and elastic net regression). The logistic regression model predicted that, on average, 58.4% of the observations were associated with the NSSI + SA group. In comparison, the proportions for the elastic net regression, random forest, and gradient boosted tree models were 58.7%, 55.2%, and 53.7%, respectively.

**Fig. 2:**
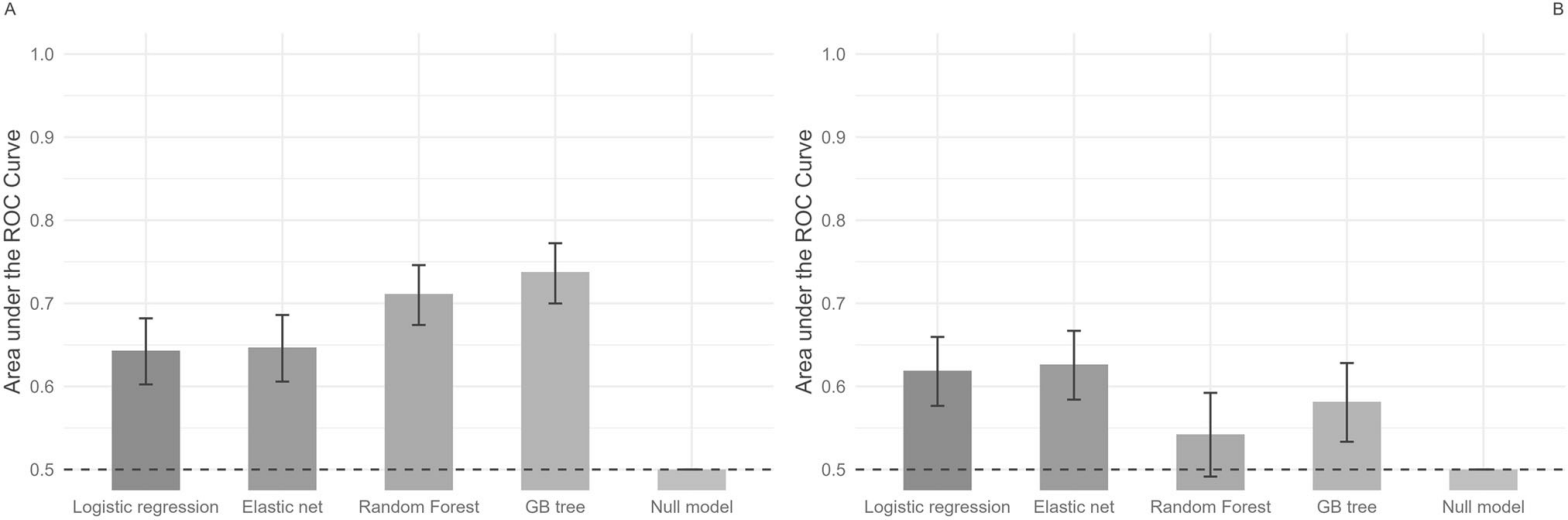
Area under the curve (AUC) for machine learning models discriminating between female patients with NSSI + SA and those with NSSI alone. Panel (**A**) includes the reduced set, depressive symptoms, and age and panel (**B**) includes only depressive symptoms, and age. The bars represent the performance of machine learning models: A model with AUC = 1 would be considered as perfect, 0.9 < AUC < 1 as excellent, 0.8 < AUC < 0.9 as good, 07 < AUC < 0.8 as fair, 0.6 < AUC < 0.7 as poor and 0.5 < AUC < 0.6 as being slightly better than chance. The error bars represent the 95% confidence intervals (CIs) of AUC values.

After adding depressive symptoms as a covariate, the performance metrics remained constant (Supplementary Fig. S1 and Supplementary Table S1). Also, controlling for age (Fig. 2A and Table 5) and sex (Supplementary Fig. S2 and Supplementary Table S2) did not change the results meaningfully.

In the models including depressive symptoms, predictors with the highest predictive value were DHEA-S and TSH. This finding was consistent across all four machine learning models (Table 4). Lower TSH and higher DHEA-S levels were associated with SA among adolescents with NSSI, although the association between DHEA-S and SA was reduced when age was included (Supplementary Table S3).

**Table 4:** Logistic regression results and variable importance for the models discriminating between female patients with NSSI + SA and those with NSSI alone using the reduced set and depressive symptoms.

Predictors	*OR*	*95% CI*	*LR importance*	*EN importance*	*GB importance*	*RF importance*
**TSH [mUl/l]**	**0.681**	**0.477–0.974**	**237/500**	**214/500**	**81/500**	**350/500**
**DHEA-S [µg/ml]**	**1.474**	**1.040–2.089**	**263/500**	**286/500**	**316/500**	**7/500**
ACTH [pg/ml]	0.968	0.928–1.009	0/500	0/500	20/500	52/500
Noradrenalin [pmol/l]	1.000	0.999–1.001	0/500	0/500	51/500	6/500
Dopamine [pmol/l]	1.000	0.999–1.001	0/500	0/500	5/500	81/500
Salivary Cortisol [nmol/l]	1.100	0.951–1.261	0/500	0/500	18/500	0/500
Depressive Symptoms	1.011	0.970–1.053	0/500	0/500	9/500	0/500
Il-6 [pg/ml]	1.035	0.917–1.168	0/500	0/500	0/500	4/500
CRP [mg/l]	1.131	0.942–1.358	0/500	0/500	0/500	0/500
(Intercept)	0.440	0.068–2.836	–	–	–	–

Importance scores (e.g., “237/500”) represent the number of times each variable was identified as the most important predictor across 500 imputed datasets for each model. For instance, “237/500” in the “LR Importance” column for TSH [mU/l] indicates that TSH was the top-ranked variable in 237 out of 500 imputations for the Logistic Regression model. These scores provide a measure of the consistency with which each variable was ranked as the most important predictor across all imputations for each model.

*OR* odds ratio, *CI* confidence interval, *CRP* C-reactive protein, *fT3* free triiodothyronine, *ACTH* adrenocorticotropic hormone, *DHEA-S* dehydroepiandrosterone-sulfate, *Il-6* Interleukin-6, *LR* Logistic Regression, *EN* Elastic Net, *GB* Gradient Boosting, *RF* Random Forest.

Bold values indicate the most important predictors across 500 imputed datasets for each model.

An additional analysis, which examined recent SA (defined as occurring within one year before baseline) as the outcome and used a reduced set of biomarkers and depressive symptoms as predictors, demonstrated an overall decrease in model performance (Supplementary Fig. S3 and Supplementary Table S4).

#### Models including only depressive symptoms and age

Machine learning models that included only depressive symptoms and age as predictors showed poor performance in distinguishing between adolescents with NSSI + SA and those with NSSI alone (Fig. 2B and Table 5). The logistic regression and elastic net models yielded AUCs slightly above chance, while the random forest and gradient boosted tree models did not perform significantly better than chance.

**Table 5:** Area under the receiver operating characteristics curve, standard deviations and confidence intervals for models discriminating between female patients with NSSI + SA and those with NSSI alone using the reduced set, depressive symptoms, and age and only depressive symptoms, and age respectively.

	Reduced set and clinical features				Clinical features only			
Model	AUC	AUCSD	LB	UB	AUC	AUCSD	LB	UB
Logistic regression	0.643	0.088	0.602	0.682	0.619	0.090	0.577	0.660
Elastic net	0.647	0.090	0.606	0.686	0.626	0.090	0.584	0.667
Random forest	0.711	0.089	0.674	0.746	0.542	0.104	0.491	0.592
Gradient-boosted tree	0.738	0.095	0.700	0.772	0.582	0.099	0.533	0.628

A model with AUC = 1 would be considered as perfect, 0.9 < AUC < 1 as excellent, 0.8 < AUC < 0.9 as good, 0.7 < AUC < 0.8 as fair, 0.6 < AUC < 0.7 as poor and 0.5 < AUC < 0.6 as being slightly better than chance. Lower (LB) and upper bounds (UB) of the 95% confidence intervals (CIs) of AUC values are reported.

## Discussion

Overall, machine learning models based on neurobiological markers showed poor to fair performance in distinguishing adolescents with NSSI + SA from those with NSSI only. Models including the reduced set of biomarkers (CRP, interleukin-6, salivary cortisol, DHEA-S, TSH, dopamine, noradrenalin, and ACTH), which are known to be associated with SA, performed better than models using the full set of markers. After including depressive symptoms, and age as covariates, performance metrics of the models including the reduced set remained constant. More complex models (random forests and gradient boosted trees) performed slightly better than simpler ones (logistic regression and elastic net regression). DHEA-S, and TSH were the predictors with the highest prediction value in discriminating between patients with NSSI + SA and those with NSSI alone. In contrast, models based solely on depressive symptoms and age performed poorly, with some complex models not exceeding chance levels.

This study effectively distinguished adolescents with NSSI + SA from those with NSSI alone using neurobiological markers, achieving accuracy above chance levels. Although model performance was moderate, these findings contribute meaningfully to suicide risk assessment and add to the evidence for a biological self-harm phenotype in adolescents [16]. Unlike previous research, our study focused on identifying those at particularly high risk within an already high-risk NSSI group. The slightly improved performance of models with a reduced biomarker set (CRP, interleukin-6, salivary cortisol, DHEA-S, TSH, dopamine, noradrenaline, and ACTH) suggests a distinct biological profile associated with SA in NSSI adolescents. This selected set, consisting solely of blood and saliva markers, enhances clinical applicability by offering a minimally invasive, cost-effective alternative to resource-intensive methods like neuroimaging or ECG.

DHEA-S and TSH may be particularly important for assessing suicide risk within this phenotype, aligning with prior studies linking these markers to SA [25, 27]. DHEA-S, a precursor to androgens and estrogens, is critical for physical and emotional development during puberty, though its association with SA appears to lessen after adjusting for age, likely due to its natural increase during adolescence [59]. Like cortisol, DHEA-S is produced in the adrenal cortex, acting as a stress response modulator, often counterbalancing cortisol [60]. Future studies should investigate the cortisol-DHEA-S interaction, as age-related changes may influence stress regulation and suicide risk.

Similarly, TSH supports the release of thyroid hormones, vital for growth, development, and mental health. Dysregulation, such as in hypothyroidism, can lead to delayed puberty, physical symptoms like fatigue, and increased risk for mood disorders—a significant suicide risk factor [26, 61]. Given the potential for thyroid dysfunction to impact both physical and mental health, further research on the HPT-axis and suicide risk could support more targeted interventions for vulnerable adolescents.

Moreover, our finding that models including the reduced set of biomarkers perform better than models using the full set underscores the significance of feature selection, a method recognized for improving learning efficiency in machine learning contexts [62]. While some models in this study inherently incorporate feature selection mechanisms (like the penalty term in elastic net regression), the results demonstrate that pre-selecting significant markers before data analysis can further enhance the accuracy of machine-learning models. Therefore, acquiring prior knowledge from research and theory might be a crucial initial step in developing algorithms for classification tasks in medical research.

Another important step in this field of research is model selection. It has been suggested, that more complex machine learning models, such as random forest and gradient boosting, might allow for the detection of nonlinear interactions between biomarkers. Our results show that complex models slightly outperform simpler ones. This implies that nonlinear interactions among biomarkers are important in differentiating patients with NSSI + SA from those with NSSI alone. Since model complexity necessitates larger sample sizes, regular clinical data collection in biobanks could enhance model performance by uncovering more complex biomarker interactions.

### Clinical implications

This study, in line with previous research, suggests that neurobiological markers could be valuable for early detection of adolescents with NSSI who are at high risk for suicide [14–16]. The integration of machine learning with neurobiological markers offers substantial potential for improving suicide prediction by identifying complex biological patterns that traditional methods may overlook. As markers of risk severity, these biological indicators could help place adolescents along a spectrum of self-harm, from less to more severe, consistent with the diathesis-stress model of suicide. According to this model, neurobiological impairments, such as HPA axis dysfunction, may play a role in mood regulation, cognitive control, and stress response, all central to suicidal behavior [63]. Including these markers in clinical assessment could therefore enhance both risk identification and intervention strategies.

Our findings also highlight that the neurobiological markers investigated may reflect a general disposition for SA rather than acute SA. Adolescents with NSSI + SA were older, took more psychoactive medication, and showed higher rates of personality disorders compared to those with NSSI alone, along with a nonsignificant tendency towards more adverse childhood experiences. These factors suggest that our models are more suited for identifying broad vulnerabilities rather than imminent risk. Furthermore, the limited predictive value of age in our models argues against a purely developmental explanation and supports the interpretation that biomarkers capture a broader vulnerability for SA.

This aligns with the stratified stepped-care model [64], where adolescents are matched to care levels based on their neurobiological risk profile and other factors. Those identified as lower risk could begin with brief, low-intensity interventions, while higher-risk adolescents would receive comprehensive treatment from the start. Over time, care could be intensified for those with persistent or worsening symptoms. This model could optimize healthcare resources and help ensure that adolescents receive care tailored to their specific risk profiles.

### Strengths and limitations

To our knowledge, this is the first study to distinguish between adolescents with NSSI + SA and those with NSSI alone using a biological phenotype. Our primary strength lies in the extensive collection of neurobiological markers and the thorough characterization of psychopathology through structured interviews, which enhances the reliability of our findings.

However, several limitations must be considered. First, the retrospective and cross-sectional nature of our study inherently restricts its ability to assess whether these models can effectively predict future SA. In contrast to longitudinal studies, our findings suggest a general disposition for SA, supported by multiple observations. Adolescents with NSSI + SA were older, took more psychoactive medication, and showed higher rates of personality disorders compared to those with NSSI alone. Additionally, there was a nonsignificant tendency for those with NSSI + SA to have experienced more adverse childhood events. These characteristics collectively indicate a broader vulnerability for SA, which may predispose individuals to SA over time, rather than reflecting acute or immediate risk. Importantly, a supplementary analysis using a more recent outcome revealed that model performance decreased. This finding reinforces the interpretation that our results point toward a general disposition for SA rather than acute SA, further underscoring the challenges of predicting imminent SA. Moreover, the lack of external validation limits the generalizability of our results despite robust internal cross-validation. Future studies should include external validation in independent cohorts to confirm model robustness and assess the clinical applicability of these biomarkers across diverse populations.

Second, we focused on selected biomarkers, so additional markers that could further differentiate between NSSI + SA and NSSI alone, such as markers derived from magnetic resonance imaging (MRI), or HPA axis reactivity, were not assessed [36, 37, 65, 66].

Third, NSSI was defined based on Criterion A of the DSM-5 research diagnosis for NSSI disorder. Additional diagnostic components (Criteria B–F) were not formally assessed. However, this approach is consistent with most current studies in the field, which primarily rely on Criterion A to identify individuals with NSSI.

Fourth, there are clinical differences between the NSSI + SA and NSSI groups; the NSSI + SA group is older and has a higher prevalence of F60 diagnoses (personality disorders), suggesting a more chronic illness course. Longitudinal studies are needed to explore how age and personality disorders affect progression from NSSI to SA.

Lastly, due to a limited number of male participants, we could not examine gender differences in SA risk. Known differences include higher rates of SA in females and higher rates of completed suicides in males [67]. Though additional analyses including 19 males did not alter our outcomes, gender-based biomarker profiles may still be relevant.

### Summary and conclusions

In conclusion, our models were able to distinguish adolescents with NSSI + SA from those with NSSI alone using biological markers. Although the performance was moderate, the findings potentially contribute to future suicide risk assessment in adolescents with NSSI. The superior performance of models utilizing selected markers (CRP, interleukin-6, salivary cortisol, DHEA-S, TSH, dopamine, noradrenaline, and ACTH) supports emerging evidence for a biological phenotype of self-harm in adolescents. TSH and DHEA-S, as primary differentiating factors, may be crucial in identifying at-risk adolescents. Future studies with longitudinal designs and advanced machine learning on larger samples could improve the identification of adolescents at varying risk levels for SA in the context of NSSI.

## Supplementary information

Supplemental Material

## Data Availability

The data that support the findings of this study are available from the corresponding author upon reasonable request. Due to ethical restrictions, individual participant data cannot be shared publicly.
