# Supplementary material for: A biological phenotype of suicide attempt in adolescents with nonsuicidal self-injury: a machine-based learning approach
Source: Neuropsychopharmacology. 2025 Jul 29;50(12):1817–26. doi: 10.1038/s41386-025-02176-2 (PMC12518856; doi:10.1038/s41386-025-02176-2)
Supplement: Supplementary file 1 — Supplemental Material [file 41386_2025_2176_MOESM1_ESM.pdf]

Supplementary materials

A biological phenotype of suicide attempt in adolescents with nonsuicidal self-injury - a machine-based learning approach

Fink et al.

Table of contents

Table of Contents

Table of contents 1

Table of tables 2
Table of figures 2
Supplementary Methods 2
Tables 3
Figures 5

Table of tables

Table S1. Area under the receiver operating characteristics curve, standard deviations and confidence intervals for models discriminating between female patients with NSSI+SA and those with NSSI alone using the reduced set and depressive symptoms.
Table S2. Logistic Regression results for the model discriminating between female patients with NSSI and SA and those with NSSI alone using the reduced set, depressive symptoms, and age.
Table S3. Area under the receiver operating characteristics curve, standard deviations and confidence intervals for models discriminating between male and female patients with NSSI+SA and those with NSSI alone using the reduced set, depressive symptoms, and age.
Table S4. Area under the receiver operating characteristics curve, standard deviations and confidence intervals for models discriminating female patients with recent SA and those without recent SA using the reduced set and depressive symptoms.

Table of figures

Figure S1. AUC for models discriminating between female patients with NSSI+SA and those with NSSI alone using the reduced set and depressive symptoms. The bars represent the performance of machine learning models: A model with AUC = 1 would be considered as perfect, 0.9 < AUC < 1 as excellent, 0.8 < AUC < 0.9 as good, 07 < AUC < 0.8 as fair, 0.6 < AUC < 0.7 as poor and 0.5 < AUC < 0.6 as being slightly better than chance. The error bars represent the 95% confidence intervals (CIs) of AUC values.
Figure S2. AUC for models discriminating between male and female patients with NSSI+SA and those with NSSI alone the reduced set, depressive symptoms, and age. The bars represent the performance of machine learning models: A model with AUC = 1 would be considered as perfect, 0.9 < AUC < 1 as excellent, 0.8 < AUC < 0.9 as good, 07 < AUC < 0.8 as fair, 0.6 < AUC < 0.7 as poor and 0.5 < AUC < 0.6 as being slightly better than chance. The error bars represent the 95% confidence intervals (CIs) of AUC values.
Figure S3. AUC for models discriminating between female patients with recent SA and those without recent SA using the reduced set, depressive symptoms, and age. The bars represent the performance of machine learning models: A model with AUC = 1 would be considered as perfect, 0.9 < AUC < 1 as excellent, 0.8 < AUC < 0.9 as good, 07 < AUC < 0.8 as fair, 0.6 < AUC < 0.7 as poor and 0.5 < AUC < 0.6 as being slightly better than chance. The error bars represent the 95% confidence intervals (CIs) of AUC values.

Supplementary Methods

<u>Reference Ranges of blood markers</u>
Reference ranges were the following: ACTH: 122-466 pg/ml; Adrenaline: <464 pmol/l; Beta endorphin: 20,5 - 121,6 pg/ml; CRP: 0.068 – 8.2 mg/l; Cortisol: 56–200 ng/ml; DHEA-S: 1,3 - 4 µg/ml; Dopamine: <560 pmol/l; Estradiol: 40.0 - 250 pg/ml; fT3: 2.0–4.2 ng/l; fT4: 8–18 ng/l; Il6: < 15 pg/ml; Leukocytes

(females 1-16 years): 4.3-13.0 /nl; Leukocytes (females 17-199 years): 4-10 /nl; Noradrenaline: <1625 pmol/l; Oxytocin: 70 - 165 pg/ml; Testosterone (females 9-12 years): 0.05-0.4 ng/ml; Testosterone (females 13-14 years): 0.08-0.5 ng/ml; Testosterone (females 15-19 years): 0.03-0.6 ng/ml; TSH: 0.4–4.0 mU/l.

Tables

Table S1. Area under the receiver operating characteristics curve, standard deviations and confidence intervals for models discriminating between female patients with NSSI+SA and those with NSSI alone using the reduced set and depressive symptoms.

Model	AUC	AUCSD	LB	UB
Logistic regression	0.628	0.090	0.586	0.669
Elastic net	0.630	0.091	0.587	0.671
Random forest	0.682	0.098	0.642	0.720
Gradient boosted tree	0.711	0.098	0.669	0.749

Note. A model with AUC = 1 would be considered as perfect, 0.9 < AUC < 1 as excellent, 0.8 < AUC < 0.9 as good, 07 < AUC < 0.8 as fair, 0.6 < AUC < 0.7 as poor and 0.5 < AUC < 0.6 as being slightly better than chance. Lower (LB) and upper bounds (UB) of the 95% confidence intervals (CIs) of AUC values are reported.

Table S2. Area under the receiver operating characteristics curve, standard deviations and confidence intervals for models discriminating between male and female patients with NSSI+SA and those with NSSI alone using the reduced set, depressive symptoms, and age.

Model	AUC	AUCSD	LB	UB
Logistic regression	0.635	0.084	0.596	0.673
Elastic net	0.638	0.085	0.600	0.677
Random forest	0.669	0.086	0.631	0.705
Gradient boosted tree	0.694	0.092	0.654	0.730

Note. A model with AUC = 1 would be considered as perfect, 0.9 < AUC < 1 as excellent, 0.8 < AUC < 0.9 as good, 07 < AUC < 0.8 as fair, 0.6 < AUC < 0.7 as poor and 0.5 < AUC < 0.6 as being slightly better than chance. Lower (LB) and upper bounds (UB) of the 95% confidence intervals (CIs) of AUC values are reported.

Table S3. Logistic Regression results for the model discriminating between female patients with NSSI and SA and those with NSSI alone using the reduced set, depressive symptoms, and age.

Predictors	***OR***	***95% CI***
**TSH [mUl/l]**	**0.650**	**0.451 – 0.936**
**Age**	**1.347**	**1.037-1.749**
DHEA-S [µg/ml]	1.300	0.892 – 1.880

ACTH [pg/ml]	0.971	0.931 – 1.012
Noradrenalin [pmol/l]	1.000	0.999 – 1.001
Dopamine [pmol/l]	1.000	0.999-1.001
Salivary Cortisol [nmol/l]	1.064	0.922-1.227
Depressive Symptoms	1.010	0.968-1.054
Il-6 [pg/ml]	1.016	0.900 – 1.151
CRP [mg/l]	1.133	0.948-1.354
(Intercept)	0.009	0.001 – 0.446

Note. A model with AUC = 1 would be considered as perfect, 0.9 < AUC < 1 as excellent, 0.8 < AUC < 0.9 as good, 07 < AUC < 0.8 as fair, 0.6 < AUC < 0.7 as poor and 0.5 < AUC < 0.6 as being slightly better than chance. Lower (LB) and upper bounds (UB) of the 95% confidence intervals (CIs) of AUC values are reported.

Table S4. Area under the receiver operating characteristics curve, standard deviations and confidence intervals for models discriminating female patients with recent SA and those without recent SA using the reduced set and depressive symptoms.

Model	AUC	AUCSD	LB	UB
Logistic regression	0.553	0.099	0.504	0.600
Elastic net	0.558	0.100	0.509	0.606
Random forest	0.610	0.100	0.562	0.655
Gradient boosted tree	0.657	0.109	0.607	0.703

Note. A model with AUC = 1 would be considered as perfect, 0.9 < AUC < 1 as excellent, 0.8 < AUC < 0.9 as good, 07 < AUC < 0.8 as fair, 0.6 < AUC < 0.7 as poor and 0.5 < AUC < 0.6 as being slightly better than chance. Lower (LB) and upper bounds (UB) of the 95% confidence intervals (CIs) of AUC values are reported.

Figures

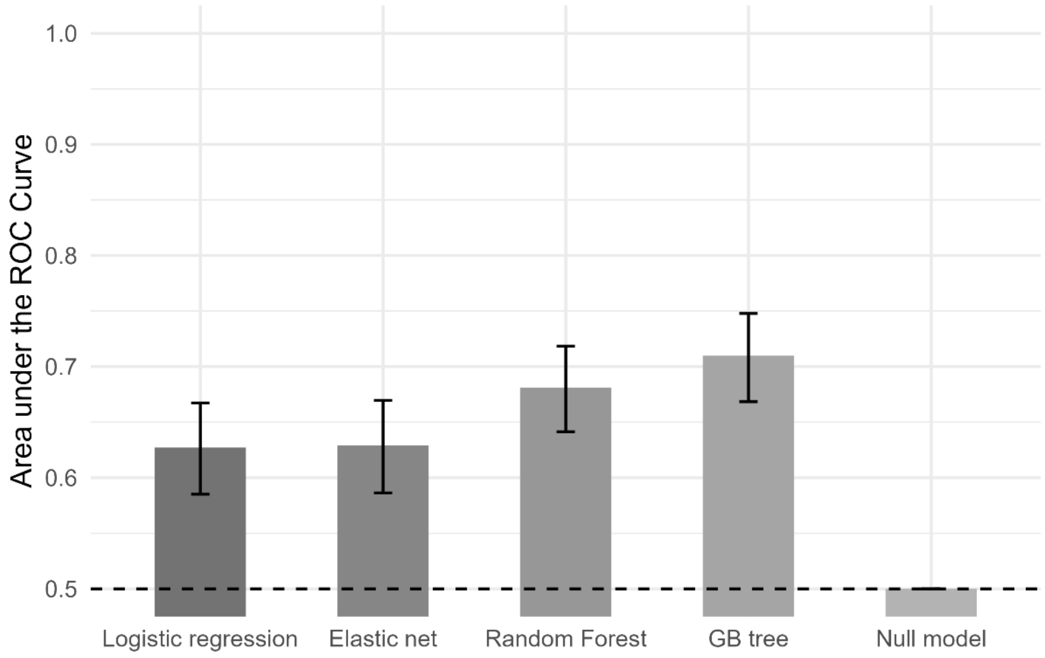

Figure S1. AUC for models discriminating between female patients with NSSI+SA and those with NSSI alone using the reduced set and depressive symptoms. The bars represent the performance of machine learning models: A model with AUC = 1 would be considered as perfect, 0.9 < AUC < 1 as excellent, 0.8 < AUC < 0.9 as good, 07 < AUC < 0.8 as fair, 0.6 < AUC < 0.7 as poor and 0.5 < AUC < 0.6 as being slightly better than chance. The error bars represent the 95% confidence intervals (CIs) of AUC values.

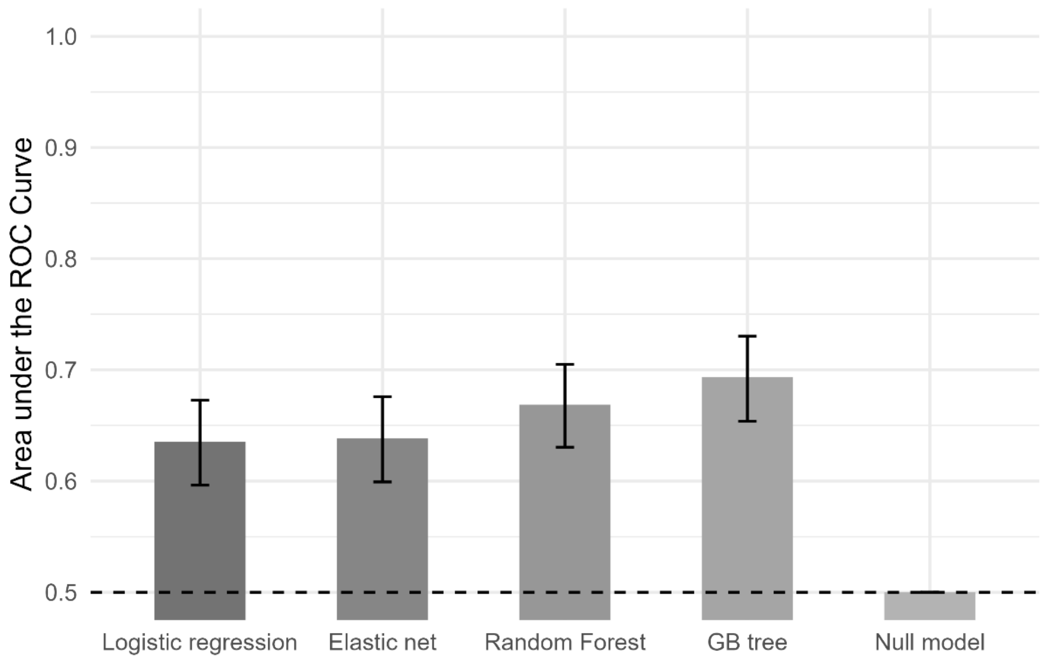

Figure S2. AUC for models discriminating between male and female patients with NSSI+SA and those with NSSI alone using the reduced set, depressive symptoms, and age. The bars represent the performance of machine learning models: A model with AUC = 1 would be considered as perfect, 0.9 < AUC < 1 as excellent, 0.8 < AUC < 0.9 as good, 07 < AUC < 0.8 as fair, 0.6 < AUC < 0.7 as poor and 0.5 < AUC < 0.6 as being slightly better than chance. The error bars represent the 95% confidence intervals (CIs) of AUC values.

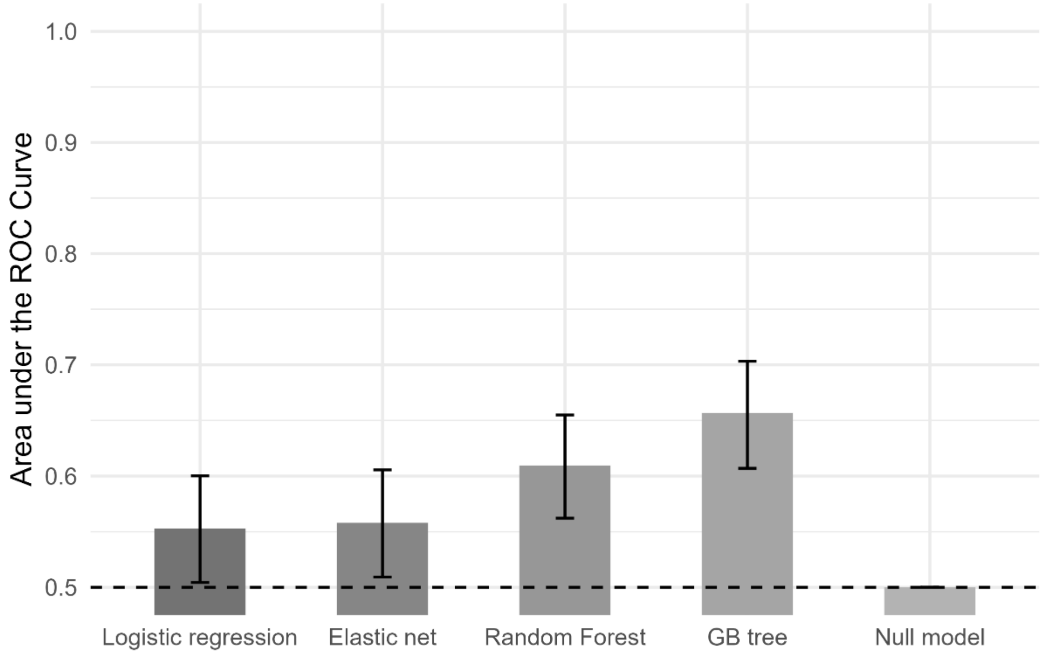

Figure S3. AUC for models discriminating between female patients with recent SA and those without recent SA using the reduced set, depressive symptoms, and age. The bars represent the performance of machine learning models: A model with AUC = 1 would be considered as perfect, 0.9 < AUC < 1 as excellent, 0.8 < AUC < 0.9 as good, 07 < AUC < 0.8 as fair, 0.6 < AUC < 0.7 as poor and 0.5 < AUC < 0.6 as being slightly better than chance. The error bars represent the 95% confidence intervals (CIs) of AUC values.
